# Reliability and Validity of Commercially Available Wearable Devices for Measuring Steps, Energy Expenditure, and Heart Rate: Systematic Review

**DOI:** 10.2196/18694

**Published:** 2020-09-08

**Authors:** Daniel Fuller, Emily Colwell, Jonathan Low, Kassia Orychock, Melissa Ann Tobin, Bo Simango, Richard Buote, Desiree Van Heerden, Hui Luan, Kimberley Cullen, Logan Slade, Nathan G A Taylor

**Affiliations:** 1 School of Human Kinetics and Recreation Memorial University St. John's, NL Canada; 2 Department of Computer Science Memorial University St. John's, NL Canada; 3 Division of Community Health and Humanities Faculty of Medicine Memorial University St. John's, NL Canada; 4 Faculty of Medicine Memorial University St. John's, NL Canada; 5 Faculty of Engineering Memorial University St. John's, NL Canada; 6 Department of Geography University of Oregon Eugene, OR United States; 7 School of Health Administration Dalhousie University Halifax, NS Canada

**Keywords:** commercial wearable devices, systematic review, heart rate, energy expenditure, step count, Fitbit, Apple Watch, Garmin, Polar

## Abstract

**Background:**

Consumer-wearable activity trackers are small electronic devices that record fitness and health-related measures.

**Objective:**

The purpose of this systematic review was to examine the validity and reliability of commercial wearables in measuring step count, heart rate, and energy expenditure.

**Methods:**

We identified devices to be included in the review. Database searches were conducted in PubMed, Embase, and SPORTDiscus, and only articles published in the English language up to May 2019 were considered. Studies were excluded if they did not identify the device used and if they did not examine the validity or reliability of the device. Studies involving the general population and all special populations were included. We operationalized validity as criterion validity (as compared with other measures) and construct validity (degree to which the device is measuring what it claims). Reliability measures focused on intradevice and interdevice reliability.

**Results:**

We included 158 publications examining nine different commercial wearable device brands. Fitbit was by far the most studied brand. In laboratory-based settings, Fitbit, Apple Watch, and Samsung appeared to measure steps accurately. Heart rate measurement was more variable, with Apple Watch and Garmin being the most accurate and Fitbit tending toward underestimation. For energy expenditure, no brand was accurate. We also examined validity between devices within a specific brand.

**Conclusions:**

Commercial wearable devices are accurate for measuring steps and heart rate in laboratory-based settings, but this varies by the manufacturer and device type. Devices are constantly being upgraded and redesigned to new models, suggesting the need for more current reviews and research.

## Introduction

Globally, physical inactivity is a pressing public health concern. A recent report suggested that about 23% of adults and 81% of school-going adolescents are not meeting physical activity guidelines [1]. Government organizations have attempted to improve these numbers by implementing initiatives aimed at promoting physical activity. Though the successful promotion of physical activity is a complex multifacetted issue, behavior change is a well-established method to increase physical activity [2]. Metrics defining physical activity guidelines from commercial wearable devices have been developed, including 10,000 steps per day [3,4] and 100 steps per minute for moderate to vigorous activity [5]. However, research has shown variation in step count among devices, and the applicability of these metrics may vary by device brand and device type [6].

Research examining consumer wearable devices, such as watches, pendants, armbands, and other accessories, is associated with various labels including Quantified Self [7] and mobile health (mHealth) [8]. These consumer wearable devices are becoming increasingly popular for purchase and use. It has been estimated that in the year 2019, 225 million consumer wearables were sold [9], and studies have suggested that more than a third of adults in Canada and Australia own and use a consumer wearable device [10,11]. Despite their popularity, research is equivocal about whether commercial wearable devices are valid and reliable methods for estimating metrics associated with physical activity including steps, heart rate, and energy expenditure.

In a recent review of 10 articles, Bunn et al [12] noted tendencies of wearables to underestimate energy expenditure, heart rate, and step count. Fitbit wearables were highly correlated with criterion measures of step count during laboratory-based assessment and had consistently high interdevice reliability for both step count and energy expenditure [13]. However, this review found that these devices tended to underestimate energy expenditure, which is consistent with a separate review of Fitbit accuracy [14] indicating that Fitbit wearables provide accurate measures only in limited circumstances.

Commercial wearable devices have the potential to allow for population-level measurement of physical activity and large-scale behavior change. However, questions remain about their reliability and validity. This is especially true of smaller and newer manufacturers of wearable devices for which few or no reliability and validity studies have been conducted. The purpose of this systematic review was to outline and summarize information about the validity and reliability of wearables in measuring step count, heart rate, and energy expenditure in any population. The information summarized herein can be used to inform consumers and can aid researchers in study design when selecting physical activity monitoring devices.

## Methods

### Design

This systematic review was conducted and reported according to the Preferred Reporting Items for Systematic Reviews and Meta-Analyses (PRISMA) guidelines [14]. The review was not registered with PROSPERO. Full-length peer-reviewed original research articles, short reports, and letters to the editor published from January 1, 2000, through May 28, 2019, were included in the search. We limited the search to articles published after the year 2000 because commercial wearable devices were not truly available before that time.

### Search Strategy

We conducted a literature search of the following databases: MEDLINE via PubMed (1946 to present); Embase (1947 to present); and SPORTDiscus with full text (1920 to present) via EBSCO. The reference lists of eligible papers were reviewed for additional pertinent references.

A librarian (KR) developed the MEDLINE search strategy, which was peer reviewed by a second librarian according to the Peer Review of Electronic Search Strategies (PRESS) 2015 Guideline Statement [15]. The MEDLINE strategy, which included Medical Subject Heading terms and text words, was translated for the other databases using database-specific controlled vocabulary. We searched the literature using multiple combinations and forms of the following key terms: *accelerometer*, *fitness tracker*, *activity monitor*, *step count*, *wearable device*, *validity*, *reliability*, *accuracy*, *Fitbit*, *Garmin*, *Misfit*, *Jawbone*, *UnderArmour*, *Samsung*, *Apple watch*, *GENEactiv*, *Empatica*, *Mio*, *Amiigo*, *Xiaomi*, *Actigraph*, *Withings*, and *Sensewear* (see Multimedia Appendix 1 for the full search strategies). An English language limit was applied. We included any abstracts and conference proceedings, as well as articles examining any population in the initial search. References were imported into EndNote X8 software (Clarivate Analytics) where duplicate references were removed. The remaining references were then imported into Covidence software (Veritas Health Innovation) for screening.

### Study Selection Strategy

The web-based systematic review software Covidence was used for this review. The titles and abstracts of the studies included from the initial database search were independently assessed by at least two authors from the team. Conflicts arising during any step of the screening for inclusion/exclusion were resolved by a third author or by consensus. Following the title and abstract screening, full-text documents of the selected studies were searched and retrieved and were independently assessed for inclusion by at least two authors (EC, JL, and DF). Any conflicts were resolved by discussion and consensus. All reviewers strictly adhered to the defined inclusion criteria.

### Eligibility Criteria

Studies that met the following criteria were included in the review: (1) use of any consumer-wearable model from the brand Apple Inc, Empatica, Fitbit, Garmin, Jawbone, Mio, Misfit, Polar, Samsung, UnderArmour, Withings, or Xiaomi; (2) specific examination of the reliability and validity measures of the aforementioned brands; and (3) examination of the device’s ability to measure a variable (step count, heart rate, or energy expenditure). Studies with fewer than 10 participants were excluded, as has been done in previous work [13]. Validity of the wearable devices was defined as follows [16]:

- Criterion validity: comparing the devices to a criterion measure of steps, heart rate, or energy expenditure.

Reliability of the trackers included the following [16]:

- Intradevice reliability: consistent test-retest results conducted within the same device.
- Interdevice reliability: consistent results across the same model of wearable device measured at the same time and worn at the same location.

The main exclusion criteria were non-English studies, opinion/magazine articles, and systematic reviews. The initial database search and title/abstract screening included articles examining the accuracy of research-grade wearable devices, but the number of returned results was unmanageable. In order to further elucidate the research question in regard to consumer-wearable devices, before full-text screening, the decision was made to exclude all studies examining the reliability and validity of research-grade devices (Actigraph, GENEactiv, Amiigo, Sensewear Armband, Yamax, Omron, Kenze Lifecorder, Digiwalker, Actical, and Actiheart). Studies in which heart rate and energy expenditure estimates were collected using a chest strap heart rate monitor and transmitted to a wearable device were also excluded. Following text screening, the decision was made to exclude abstracts and conference papers. Following data extraction, the decision was made to exclude all studies examining Jawbone commercial wearables, as the company’s application program interface (API) was taken offline in 2018, rendering associated devices defunct. Studies were included in the final review if they had extractable data for the following criterion validity measures: correlation coefficient, group mean or percentage difference, median or mean absolute percentage error (MAPE), or level-of-agreement analysis, or had correlation coefficients for reliability measures. Authors were not contacted if these data were not reported in published or supplementary material. The remaining articles were those that met the inclusion criteria (consumer-grade wearables).

### Risk of Bias

In our risk of bias assessment, comparisons that did not report group percentage differences or correlation coefficients (n=192) were excluded from the quantitative analysis. However, rather than exclude these comparisons and studies from the review completely, we included them in a narrative summary of how the measures reported were or were not consistent with exploration of percentage measurement error and correlation.

### Data Extraction

We first conducted and documented an in-depth web search of the available consumer-wearable models and their specifications (placement, size, weight, cost, and connectivity). The data extraction process then consisted of the following: (1) categorizing the selected full-text articles into reliability or validity studies (EC, JL, and DF); (2) using a modification of the modified Consensus-Based Standards for the Selection of Health Status Measurement Instruments (COSMIN) validation subscale used by Feehan et al [13] and an a priori modified COSMIN reliability subscale (Multimedia Appendix 2) to assess the quality and risk of bias of each study (EC and DF); (3) extracting the key characteristics from each selected publication and compiling them into tables. Details from each reviewer were compared, and inconsistencies were resolved through consensus before compiling the results (EC and DF).

Data extracted included characteristics of studies, participants, and devices, including study setting and activity type, outcomes measured, and type of criterion measure used. Correlation coefficients were extracted for all reliability comparisons reported in each study. Correlation coefficients, percentage difference and group mean values, MAPE values, and level-of-agreement data were extracted for all validity comparisons where available. Where group percentage differences were not reported, we calculated group percentage error ([wearable_mean_ – criterion_mean_]/criterion_mean_ × 100) to allow for comparison across studies. We split a small number of studies (n=10) into “substudies” (n=21), where separate populations were examined in the same publication (see Multimedia Appendix 3 for a more detailed breakdown).

### Syntheses

Given the wide range of testing conditions and reported outcomes, we were unable to conduct meta-analyses of the extracted data. We instead conducted a narrative synthesis of the available quantitative data within each examined measure (step count, heart rate, and energy expenditure) using correlation comparisons and group percentage difference as the common metrics for criterion validity and correlation coefficient as the common metric for reliability.

Our interpretation of measurement accuracy was focused on acceptable limits of percentage difference of ±3% in controlled settings and percentage difference of ±10% in free-living settings, as outlined in previous work [13]. We interpreted correlation coefficients as follows: 0 to <0.2, very weak; ≥0.2 to <0.4, weak; ≥0.4 to <0.6, moderate; ≥0.6 to <0.8, strong; and ≥0.8 to 1.0, very strong [17]. We completed all quantitative analyses and plots using RStudio version 1.2.1335 (RStudio Inc) and R version 3.6.0 (The R Foundation).

Secondary analyses explored device brand. Brands were only included in these analyses when the group had 10 or more comparisons available for the measure. Studies that did not report data allowing for the examination of group percentage measurement error were still included in the review if they reported level of agreement or MAPE data. Such studies were included in the risk of bias assessment, the synthesis of study characteristics, and the narrative synthesis of study results.

### Availability of Data and Materials

Data are publicly available on the BeapLab Dataverse [18], and the analysis code is available on Github [19].

## Results

**Figure 1:**
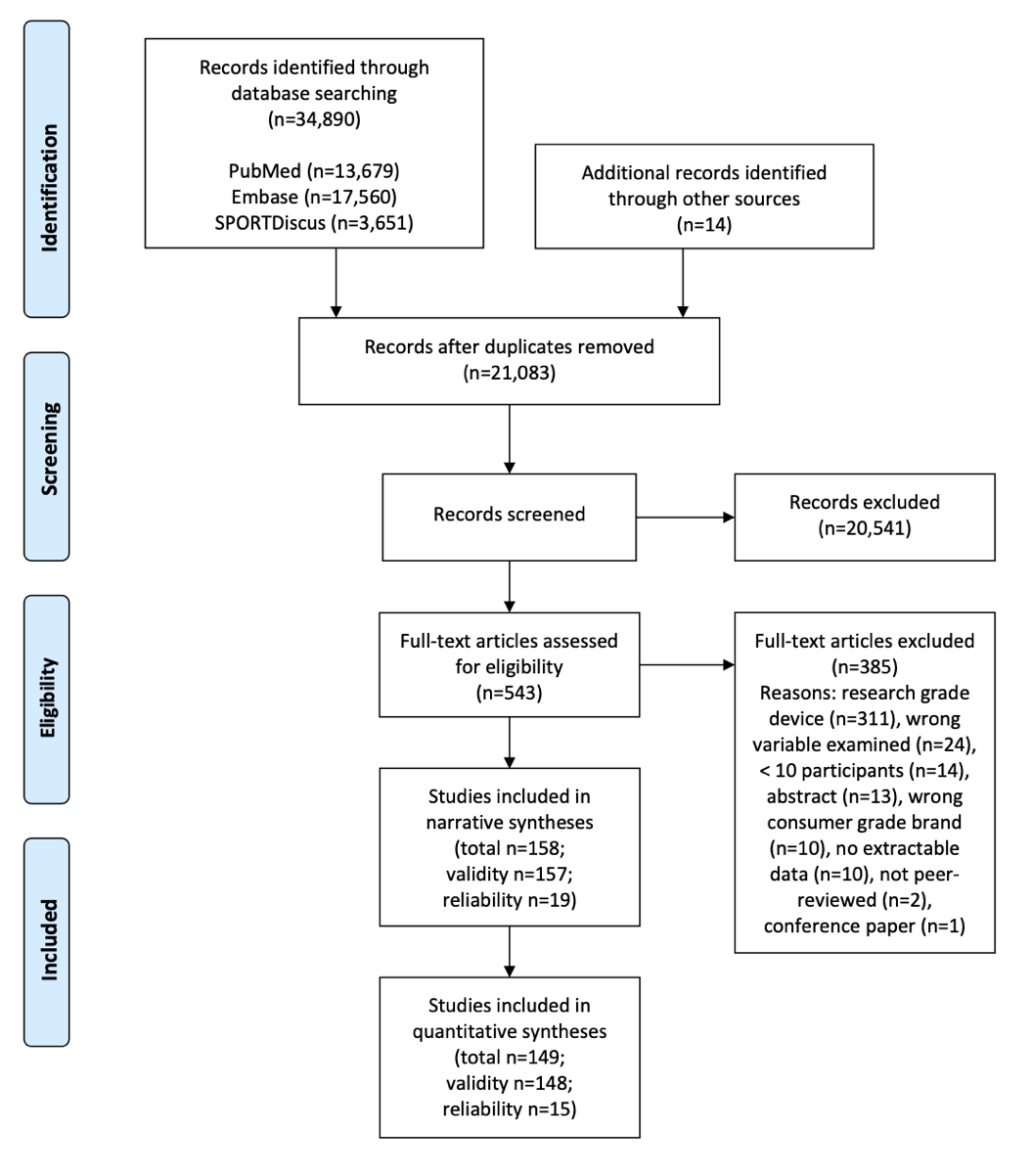
PRISMA flow chart for systematic review of the reliability and validity of commercial wearable devices.

The initial literature search from the three databases yielded 34,890 unique citations (13,679 [39.21%] from PubMed, 17,560 [50.33%] from Embase, and 3651 [10.46%] from SPORTDiscus). Fourteen additional records were identified through other sources (eg, article reference lists and social media). After duplicate references were removed, 21,083 citations remained. Based on the subsequent title and abstract screening, 20,541 were rejected because they did not meet the inclusion criteria or met the exclusion criteria. Of the 542 that remained for full-text screening, 385 (71.0%) were further excluded for the following reasons: research-grade devices (n=311, 57.4%), wrong variable examined (n=24, 4.4%), fewer than 10 participants (n=14, 2.6%), abstracts (n=13, 2.4%), wrong consumer-grade brand examined (n=10, 1.9%; devices were Yamax, Omron, Kenz Lifecorder, Digiwalker, and uniaxial Actical/Actiheart), no extractable data (n=10, 1.9%), not peer reviewed (n=2, 0.4%), and conference paper (n=1, 0.2%). As a result, a total of 158 publications were included in this systematic review (Figure 1) [14]. Table 1 shows the details of the device brand, model, year, and status (current model or discontinued) in the included studies.

**Table 1:** Device brand, model, year, current status, wear location, and studies used for the current systematic review.

Brand	Model	Year	Status	Wear location	Studies
Apple	Watch	2015	Discontinued	Wrist	[20-40]
Apple	Watch Series 2	2016	Discontinued	Wrist	[41-44]
Fitbit	Alta	2016	Current model	Wrist	[45]
Fitbit	Blaze	2016	Discontinued	Wrist	[22,40,43]
Fitbit	Charge	2014	Discontinued	Wrist	[45-56]
Fitbit	Charge 2	2016	Discontinued	Wrist	[23,30,43,44,57-63]
Fitbit	Charge HR	2015	Discontinued	Wrist	[20,21,29,32,34,36,38,45,53,64-82]
Fitbit	Classic	2009	Discontinued	Ankle/foot or waist/hip	[83-87]
Fitbit	Flex	2013	Discontinued	Thigh or wrist	[45,50,72,79,80,88-117]
Fitbit	Flex 2	2017	Current model	Wrist	[113]
Fitbit	Force	2013	Discontinued	Wrist	[118,119]
Fitbit	One	2012	Discontinued	Ankle/foot, pant pocket, waist/hip, or wrist	[34,49,52,73,80,88,90,92,93,98,100,102,103,110, 116-118,120-138]
Fitbit	Surge	2015	Discontinued	Wrist	[27,35,42,45,54,82,139-143]
Fitbit	Ultra	2011	Discontinued	Chest, pant pocket, upper arm, waist/hip, or wrist	[85,144-148]
Fitbit	Zip	2012	Current model	Ankle/foot, pant pocket, shin, or waist/hip	[34,45,46,51,88,89,92,93,96,103,112,119,127,129, 131,141,149-161]
Garmin	Fenix 3 HR	2016	Discontinued	Wrist	[41]
Garmin	Forerunner 225	2015	Discontinued	Wrist	[21,162]
Garmin	Forerunner 235	2015	Current model	Wrist	[40,139,163]
Garmin	Forerunner 405CX	2009	Discontinued	Wrist	[164]
Garmin	Forerunner 735XT	2016	Current model	Wrist	[35]
Garmin	Forerunner 920XT	2014	Discontinued	Wrist	[53]
Garmin	Vivoactive	2015	Discontinued	Wrist	[53]
Garmin	Vivofit	2014	Discontinued	Wrist	[35,46,50,52,53,89,92,104,114,122,130,143,150,159, 165-169]
Garmin	Vivofit 2	2015	Discontinued	Wrist	[34,127,170]
Garmin	Vivofit 3	2016	Discontinued	Wrist	[168,171]
Garmin	Vivosmart	2014	Discontinued	Wrist	[32,53,75]
Garmin	Vivosmart HR	2015	Discontinued	Wrist	[36,43,65]
Garmin	Vivosmart HR+	2016	Current model	Wrist	[58,63,140]
Mio	Alpha	2013	Discontinued	Wrist	[25,38,71]
Mio	Fuse	2015	Discontinued	Wrist	[54,64]
Misfit	Flash	2015	Discontinued	Waist/hip	[32]
Misfit	Shine	2012	Discontinued	Ankle/foot, chest, pant pocket, waist/hip, or wrist	[74,79,89,96,99,104,105,131,159,169]
Polar	A300	2015	Discontinued	Wrist	[172]
Polar	A360	2015	Discontinued	Wrist	[25,43,140]
Polar	Active	2011	Discontinued	Wrist	[173]
Polar	Loop	2013	Discontinued	Wrist	[32,50,53,79,89,167]
Polar	M600	2016	Current	Wrist	[56]
Polar	V800	2016	Discontinued	Wrist	[174]
Samsung	Gear 2	2014	Discontinued	Wrist	[140]
Samsung	Gear S	2014	Discontinued	Wrist	[32,38]
Samsung	Gear S2	2015	Discontinued	Wrist	[35]
Samsung	Gear S3	2016	Discontinued	Wrist	[42,44]
Withings	Pulse	2013	Discontinued	Collar, pant pocket, waist/hip, or wrist	[89,96,131,166]
Withings	Pulse O2	2013	Discontinued	Collar, waist/hip, or wrist	[104,122,123,169,175]
Withings	Pulse Ox	2014	Current model	Waist/hip or wrist	[53,58]
Xiaomi	Mi Band	2014	Discontinued	Wrist	[42]
Xiaomi	Mi Band 2	2016	Discontinued	Wrist	[81]

### Study and Participant Characteristics

Of the 158 publications included, 143 were full-text research articles, 10 were brief reports, and five were letters to the editor. Publication year ranged from 2013 to 2019, with the amount of publications increasing from 2013 to 2017 (2013, n=2; 2014, n=8; 2015, n=11; 2016, n=30; 2017, n=43). We also included an additional 40 and 24 studies published in 2018 and 2019, respectively.

**Figure 2:**
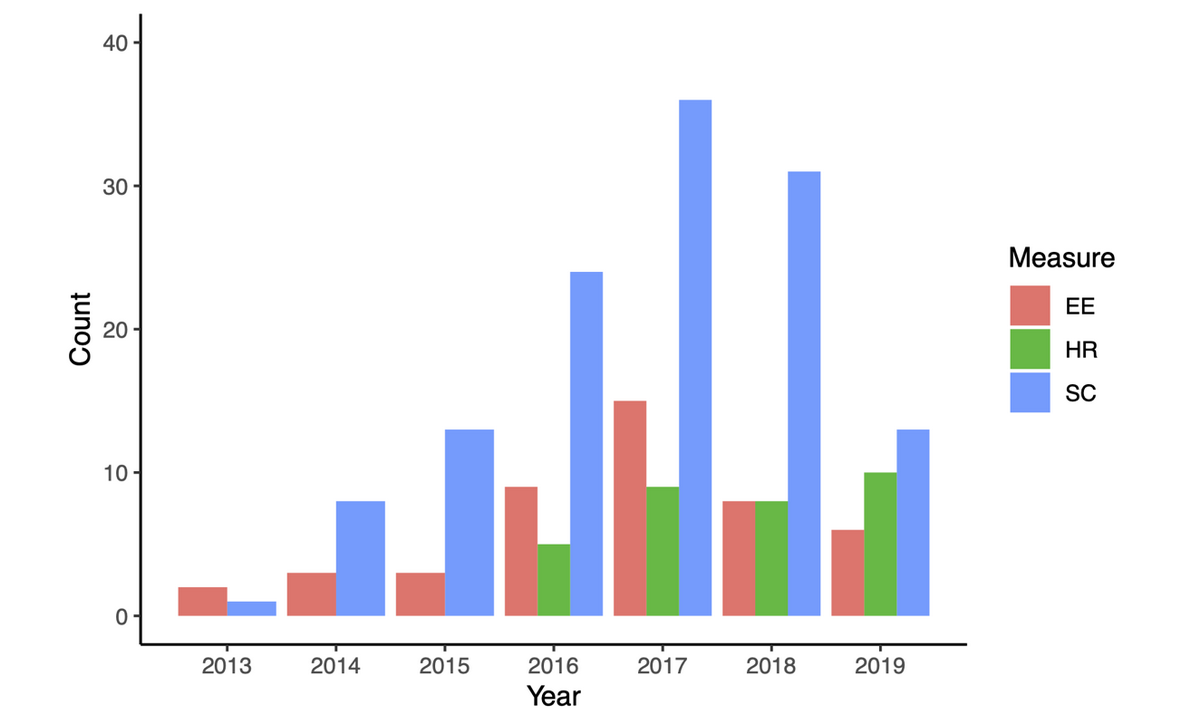
Number of studies published per year by measurement type. EE: energy expenditure; HR: heart rate; SC: step count.

Within those 158 publications, 169 studies/substudies were identified. Among these, 168 (99.4%) examined validity and 19 (11.2%) examined reliability. Moreover, 126 studies examined step count (125 validity and 16 reliability), 32 examined heart rate (32 validity and 3 reliability), and 43 examined energy expenditure (42 validity and 5 reliability) (Figure 2). Furthermore, 130 examined populations in a controlled environment and 48 examined populations in a free-living environment. A total of 1838 comparisons were identified, of which 166 examined reliability (mean 8, SD 11 per reliability study; range 1-40) and 1672 examined validity (mean 10, SD 15 per validity study; range 1-98).

The 169 studies/substudies comprised a total of 5934 participants, with a mean of 35 (SD 27) participants per study (range 10-185). One hundred and sixty-one studies reported sex, and 51.08% (2861/5601) of participants were female. One hundred and fifty-eight studies reported age, with a mean participant age of 36.8 years (SD 18.3; range 3.7-87 years). One hundred and fifty-nine studies examined adult populations (age ≥18 years) and 10 studies examined children. One hundred and thirty-three studies included only healthy participants, while the other 36 studies included participants with mobility limitations and/or chronic diseases (Multimedia Appendix 4).

**Figure 3:**
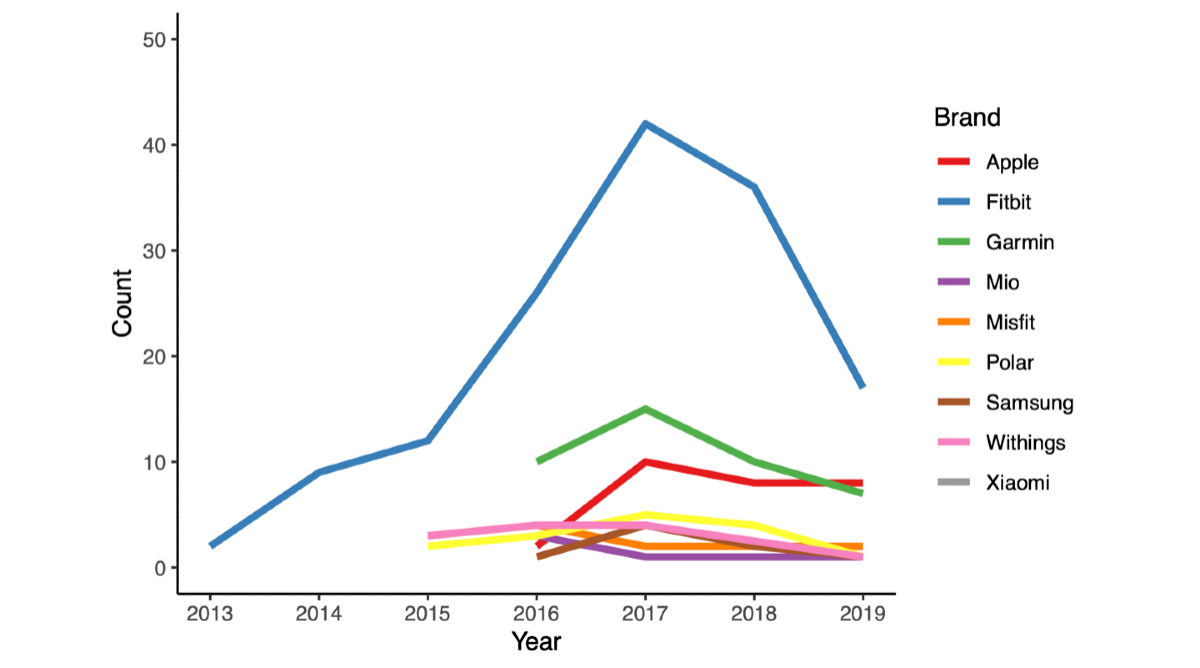
Line graph of studies published per year by device brand.

Fitbit consumer-grade wearables were examined most frequently (144 studies examining 12 models), followed by Garmin (42 studies, 13 models), Apple (28 studies, 2 models), Polar (15 studies, 6 models), Misfit (13 studies, 2 models), Withings (12 studies, 2 models), Samsung (8 studies, 4 models), Mio (6 studies, 2 models), and Xiaomi (2 studies, 2 models) (a complete list of examined models is provided in Multimedia Appendix 5) (Figure 3). Wearables were typically examined while worn on the wrist (n=131, examining at least one wrist-worn device) or at the waist/hip (n=71, locations included the waist, hip, belt, and pants pocket). Substantially fewer studies examined wearables worn on the torso (n=14, locations included the chest, bra, lanyard, and shirt collar) and lower limb (n=13, locations included the thigh, shin, ankle, and foot).

### Risk of Bias

**Figure 4:**
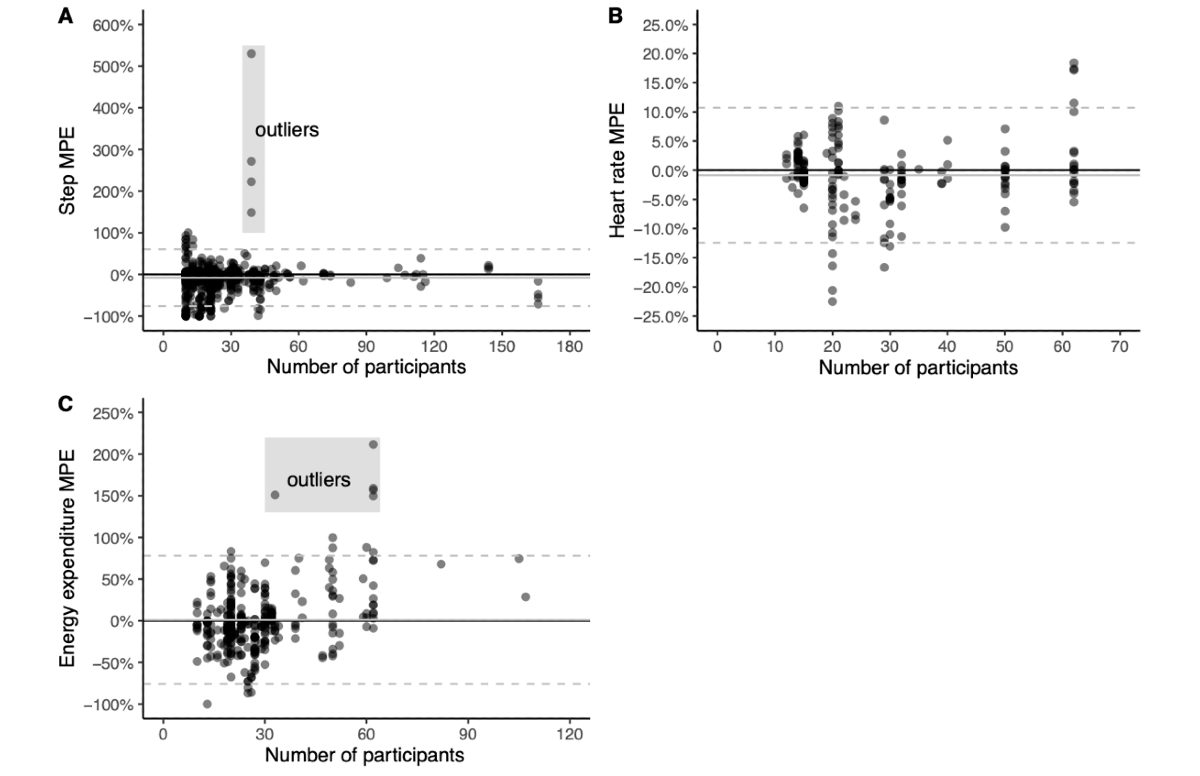
Mean percentage error (MPE) plots by study sample size for step count, heart rate, and energy expenditure. The solid black line represents zero. The solid grey line represents average MPE for all data points. The dashed grey lines represent the 95% CIs.

Of 169 studies, 140 (82.8%; 1640 of 1838 [89.23%] comparisons) were rated fair or poor for sample size (<50 participants), but were not excluded from the analysis owing to the paucity of studies with excellent (≥100 participants, n=7) and good (50-99 participants, n=22) sample sizes. We additionally explored the potential for bias related to sample size in step count, heart rate, and energy expenditure by examining the percentage error dispersion by sample size using scatter plots (Figure 4).

In these examinations, we saw no apparent systematic bias for measurement error beyond a small number of comparisons showing extreme overestimation (four comparisons in step count and five comparisons in energy expenditure). The four extreme outliers for step count involved measurement during sedentary and light physical activity in a single study with fewer than 40 participants [20] and were likely inflated by the limited number of steps accumulated during those bouts. As a result, we excluded these four comparisons from the quantitative syntheses. Upon closer examination of the five extreme outliers for energy expenditure (four occurred in a study with greater than 60 participants [21] and one occurred in a study with fewer than 40 participants [41]), we determined that these were likely true reflections of tendencies to overestimate energy expenditure during sedentary and low-intensity activities, and therefore, we included these five comparisons in the quantitative syntheses.

### Validity: Controlled Settings

We examined criterion validity for step count, heart rate, and energy expenditure separately for controlled and free-living settings. For controlled settings, we also had sufficient data to examine validity by brand and devices within brands.

#### Validity for Step Count in Controlled Settings

A total of 90 studies (979 comparisons) examined wearable device step count measurements compared with reference standard criterion measures of manual counting [32,34-38,42,46,47,50-53,57,58,72,80-84,88-102,109, 114-125,138-141,144-147,149-153,158-161,165,169-171,173] and accelerometry [20,60,64-66,85,103,109,126-128,148, 154,164] (Multimedia Appendix 6). Of these, 67 studies recruited healthy adults (mean age 35.4 years, SD 17.4 years), 20 studies recruited adults living with limited mobility/chronic diseases (mean age 60.1 years, SD 10.5 years), two studies recruited children living with limited mobility/chronic diseases (mean age 12.5 years, SD 2.9 years), and one study recruited healthy children (mean age 3.7 years, SD 0.6 years). Wearable devices were worn on the lower limb (foot, ankle, shin, and thigh), torso, waist/hip, and wrist.

Group measurement error was reported or calculable for 805 of the 979 comparisons, regardless of the criterion measure. Of these, 45.2% (n=364) were within ±3% measurement error, 42.7% (n=344) were below −3% measurement error, and 12.1% (n=97) were above 3% measurement error. The overall tendency was to underestimate step count (mean: −9%, median: −2%).

#### Validity for Heart Rate in Controlled Settings

A total of 29 studies (266 comparisons) examined wearable device heart rate measurements compared with reference standard criterion measures, including electrocardiography [22,23,38-40,43,44,54,61,62,67-70,142,162,176], Polar brand chest straps [20,21,24-28,58,63,71,163], and pulse oximetry [66], in controlled settings (a detailed list of the criterion measures used is presented in Multimedia Appendix 6). Of these, 24 studies recruited healthy adults (mean age 29.8 years, SD 10.5 years), four studies recruited adults living with limited mobility/chronic diseases (mean age 59.6 years, SD 9.0 years), and one study recruited children undergoing surgery (mean age 8.2 years, SD 3.1 years). All wearable devices were worn on the wrist.

Group measurement error was reported or calculable for 177 of 266 comparisons, regardless of the criterion measure. Of these, 56.5% (n=100) were within ±3% measurement error, 24.9% (n=44) were below −3% measurement error, and 18.6% (n=33) were above 3% measurement error. There was a slight overall tendency toward underestimation of heart rate (estimated median error: −1%).

#### Validity for Energy Expenditure in Controlled Settings

A total of 36 studies (312 comparisons) examined wearable device energy expenditure measurements compared with reference standard criterion measures, including direct calorimetry [86,104] and indirect calorimetry [20,21,29-31,38,39,41-43,53,55,63,66,73,85,87,93,95,97,103, 105,116,117,129,130,142,143,146,148,159,165,166,177], in controlled settings. Of these, 35 studies recruited healthy adults (mean age 27.2 years, SD 7.1 years), and one study recruited adults living with cardiovascular disease (mean age 64.2 years, SD 2.3 years). Wearable devices were worn on the wrist, waist/hip, and torso.

Group measurement error was reported or calculable for 305 of the 312 comparisons, regardless of the criterion measure. Of these, 9.2% (n=28) were within ±3% measurement error, 54.1% (n=165) were below −3% measurement error, and 36.7% (n=112) were above 3% measurement error. Studies showed a tendency to underestimate energy expenditure and to provide inaccurate measures of energy expenditure compared with the criterion.

### Validity in Controlled Settings by Brand

**Figure 5:**
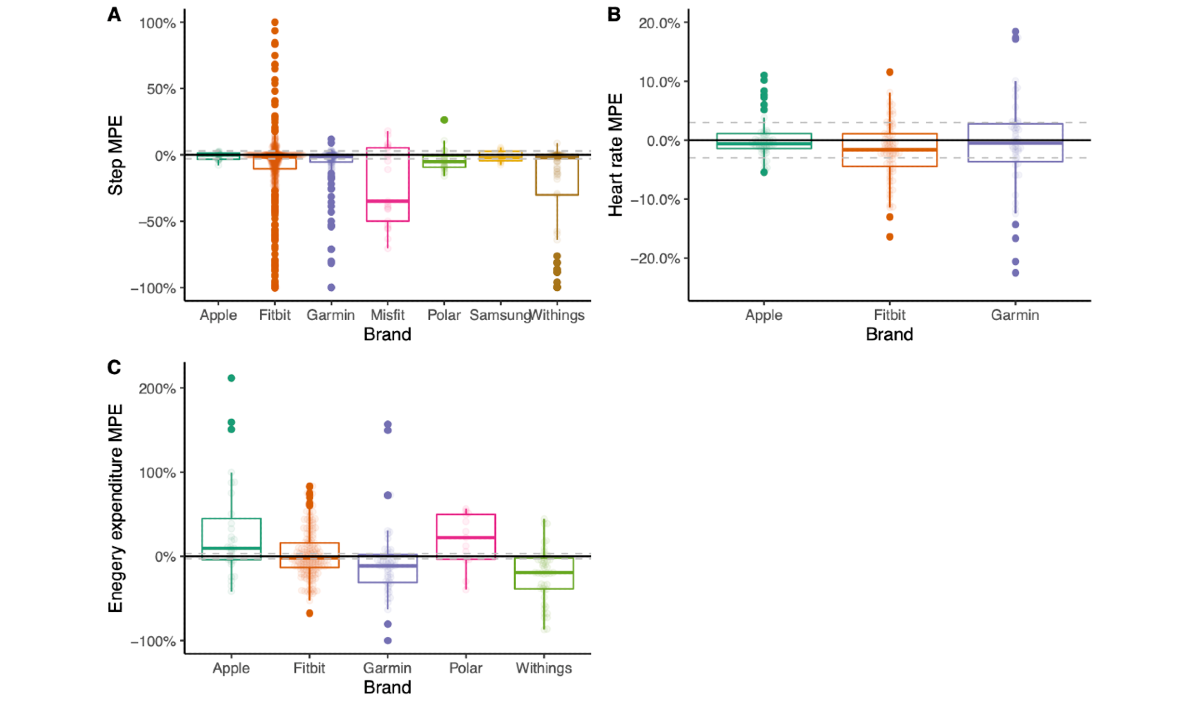
Box plots representing mean percentage error (MPE) for steps, heart rate, and energy expenditure by device brand for devices with 10 or more comparisons.

**Figure 6:**
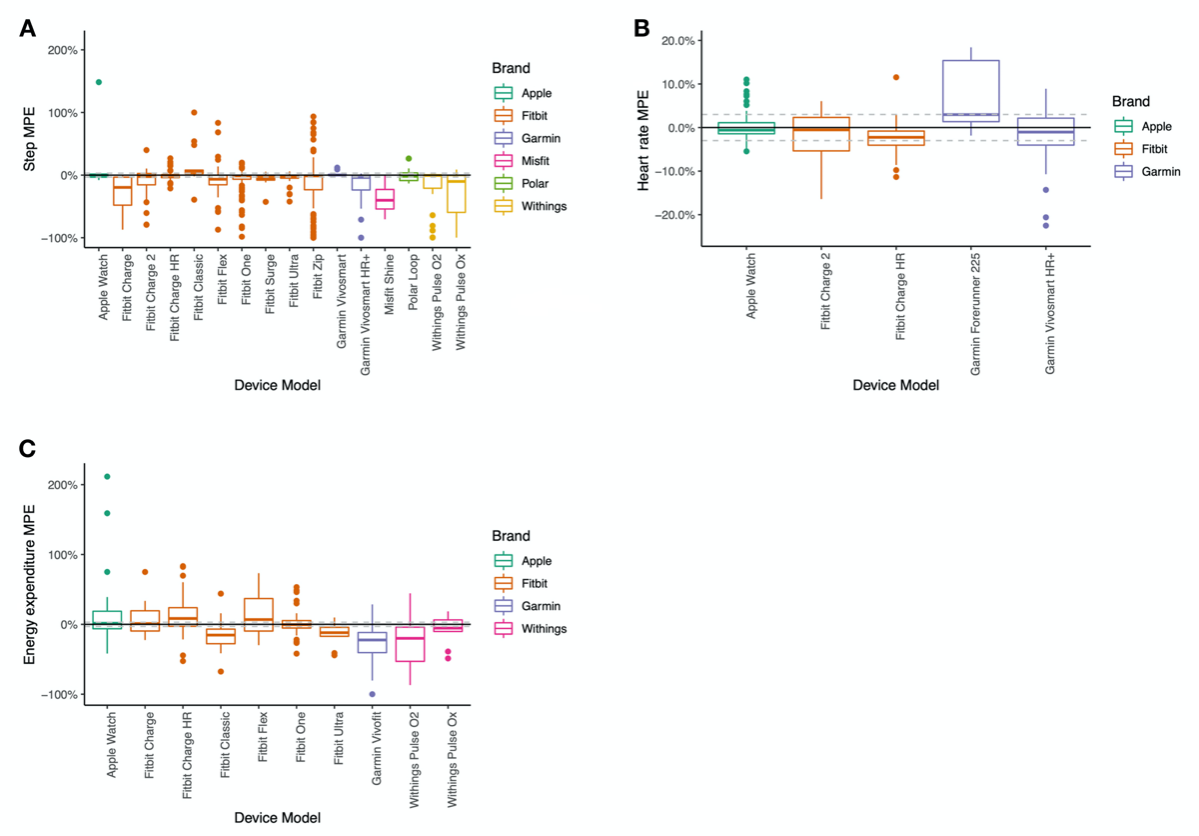
Box plots representing mean percentage error (MPE) for steps, heart rate, and energy expenditure by device brand and model for devices with 10 or more comparisons.

Figure 5 shows the mean percentage error (MPE) for step count, heart rate, and energy expenditure by device brand for devices with 10 or more comparisons. Figure 6 shows the MPE for step count, heart rate, and energy expenditure by device brand and model for devices with 10 or more comparisons.

#### Validity for Step Count by Brand

We observed that the error level varied by device brand (Figure 5). Withings and Misfit wearables consistently underestimated step count, and Apple and Samsung had less measurement variability than other brands. There are possible interactions between the number and size of studies and device wear location that may influence the brand comparisons. For example, Apple Watch and Samsung have the tightest ranges for step count estimates but have relatively fewer studies compared with other brands.

#### Validity for Heart Rate by Brand

For heart rate, measurement error also varied by device brand (Figure 5). Apple Watch was within ±3% 71% (35/49) of the time, while Fitbit wearables were within ±3% 51% (36/71) of the time and Garmin wearables were within ±3% 49% (23/47) of the time. Despite similar ±3% measurement error rates, Fitbit appeared to underestimate heart rate more than Apple Watch and Garmin.

#### Validity for Energy Expenditure by Brand

For energy expenditure estimates, no brand of wearable was within ±3% measurement error more than 13% of the time (Figure 5). Underestimation of energy expenditure (less than −3%) was observed in Garmin wearables 69% (37/51) of the time and in Withings wearables 74% (34/46) of the time. Conversely, Apple wearables overestimated energy expenditure 58% (18/31) of the time and Polar wearables overestimated energy expenditure 69% (9/13) of the time. Fitbit devices tended to provide inaccurate measures compared with the criterion, underestimating 48.4% (76/157) of the time and overestimating 39.5% (62/157) of the time, despite the boxplot in Figure 5 showing a reasonable median value for accuracy.

### Validity: Free-Living Settings

There were relatively few studies on wearable device validity in free-living conditions. Fitbit was the only brand with more than 10 studies published for step count validity in free-living conditions, and no brands had more than 10 studies for heart rate or energy expenditure. As a result, we have not shown plots of MPE for free-living conditions.

#### Validity for Step Count in Free-Living Settings

A total of 42 studies (84 comparisons) examined wearable device step count measurements compared with the reference standard criterion measure of accelerometry [33,45,48,49, 56,59,60,64,74-76,89,96,101,106-112,120,131-136, 149,154-156,159,167,168,172-175] in free-living settings (Multimedia Appendix 6). Of these, 28 studies recruited healthy adults (mean age 33.7 years, SD 13.9 years), nine studies recruited adults living with limited mobility/chronic diseases (mean age 60.1 years, SD 11.2 years), four studies recruited healthy children (mean age 12.5 years, SD 2.6 years), and one study recruited children living with cardiac diseases (mean age 13 years, SD 2.2 years). Wearable devices were worn on the lower limb (foot, ankle, and shin), torso, waist/hip, and wrist.

Group measurement error was reported or calculable for 69 of the 84 comparisons, regardless of the criterion measure. Of these, 42% (n=29) were within ±10% measurement error, 17% (n=12) were below −10% measurement error, and 41% (n=28) were above 10% measurement error. The overall tendency was slight overestimation of step count (mean: 5%, median: 6%). Among the remaining comparisons, 11 of 15 reported MAPE, of which 40% (n=6) were below 10% measurement error and 60% (n=9) were above 10% measurement error.

#### Validity for Heart Rate in Free-Living Settings

Three studies (five comparisons) examined wearable device heart rate compared with the reference standard criterion measure of a Polar brand chest strap in free-living settings [75,77,78]. Of these, one study recruited healthy adults (mean age 25.4 years, SD 3.7 years), one study recruited healthy children (mean age 8 years, SD 1.8 years), and one study recruited adults recovering from stroke (mean age 64.4 years, SD 15 years). All wearable devices were worn on the wrist. Group measurement error was reported or calculable for one of the five comparisons, with the Fitbit Charge HR falling within ±10% measurement error in the study examining healthy children. Three of the four remaining comparisons examined the Fitbit Charge HR in adults and noted underestimation of heart rate that varied depending on activity intensity, but all reported that MAPE values fell within 10% measurement error. Correlation coefficients were strong to very strong in four of the five comparisons and moderate in one comparison examining estimation during high-intensity activity.

#### Validity for Energy Expenditure in Free-Living Settings

Nine studies (22 comparisons) examined energy expenditure in free-living settings compared with the criterion measures of doubly labeled water [104] and accelerometry [29,49,79,101,131,172,174,175]. Eight studies recruited healthy adults (mean age 27.7 years, SD 3.8 years) and one study recruited adults with chronic obstructive pulmonary disease (mean age 66.4 years, SD 7.4 years). Wearable devices were worn on the wrist or waist/hip.

Group measurement error was reported or calculable for 17 of the 22 comparisons, regardless of the criterion measure. Of these, 18% (n=3) were within ±10% measurement error, 53% (n=9) were below −10% measurement error, and 29% (n=5) were above 10% measurement error. There was an overall tendency to underestimate energy expenditure (mean: −3%, median: −11%). Xiaomi data were not analyzed in a single indirect calorimetry study owing to the lack of data [53].

### Reliability

Nineteen studies (166 comparisons) with sample sizes ranging from 11 [94] to 56 [151] reported inter- or intradevice reliability for Apple (seven comparisons), Fitbit (92 comparisons), Garmin (22 comparisons), Polar (one comparison), and Withings (44 comparisons). The majority of comparisons (153/166) reported interdevice reliability for step count, heart rate, or energy expenditure. No studies reported intradevice reliability for heart rate or energy expenditure. We have not reported between-brand comparisons for inter- or intradevice reliability owing to the small number of comparisons for each brand.

#### Interdevice Reliability for Step Count

Twelve studies (51 comparisons) with sample sizes ranging from 13 [117,138] to 56 [151] reported on interdevice reliability for step count [50,58,72,85,94,110,113,116,117,121,125, 138,151,161,171]. The majority of correlation coefficients for step count interdevice reliability were very strong (n=35), with only a small number (n=3) being reported as strong.

#### Intradevice Reliability for Step Count

Two studies (13 comparisons) reported on intradevice reliability for step count, with sample sizes of 20 [82] and 24 [150]. Intradevice reliability correlations were very weak (n=1), weak (n=2), moderate (n=5), strong (n=2), and very strong (n=3). The mean correlation coefficient was 0.58.

#### Interdevice Reliability for Heart Rate

Three studies (23 comparisons) examined interdevice reliability for heart rate [24,26,58], with analyzed sample sizes ranging from 13 [24] to 21 [26]. Apple Watch showed very good interdevice reliability at 5-s epochs during treadmill bouts at 4, 7, and 10 km/h, with reliability increasing and standard typical error decreasing with increasing pace [26]. Similar standard typical error levels were seen in maximum heart rate measured during a single incremental maximal oxygen uptake test performed on a treadmill and heart rate taken from the highest 30-s mean heart rate, with somewhat lower correlation coefficients [24]. In the examination of interdevice reliability in healthy older adults, Fitbit Charge 2 showed good reliability during treadmill and overground bouts and poor reliability during hand movement tasks such as dusting [58]. During the same tasks, Garmin Vivosmart HR+ showed good reliability during all tasks and had narrower limits of agreement than Fitbit.

#### Interdevice Reliability for Energy Expenditure

Five studies (50 comparisons) reported on interdevice reliability [85,113,116,117,166], with analyzed sample sizes ranging from 13 [117] to 29 [113]. All five studies recruited healthy adults (mean age 26.3 years, SD 3.9 years). Correlation coefficients were reported for 16 of 50 comparisons. Of these, 13% (n=2) were rated very weak, 6% (n=1) were rated moderate, 6% (n=1) were rated strong, and 75% (n=12) were rated very strong.

## Discussion

### Overview

The purpose of this study was to examine the validity and inter- and intradevice reliability of commercial wearable devices in measuring steps, heart rate, and energy expenditure. Our review focused on both a breadth of devices and reproducibility. Our review included nine brands and 45 devices with the number of comparisons ranging from 201 for the Fitbit Zip to one for the Garmin Forerunner 405CX and the Polar M600. For comparison, two recent reviews from 2017 included two brands and 16 devices [13] and seven brands and eight devices [79]. A review from 2016 included eight devices [32]. Along with this review, we have published our dataset and code to reproduce our findings.

Our bias assessment showed no apparent bias toward studies of different sample sizes. However, there is a strong overrepresentation of studies with 20 participants. There were some outliers in our findings; however, considering the number of included comparisons, this is to be expected.

### Reliability and Validity

Criterion validity of commercial wearables varied by study type (controlled or free-living), brand, and device. For step count, our review showed that in controlled laboratory settings, a higher proportion of devices showed accuracy, and this was within a tighter limit of acceptable accuracy compared with free-living conditions. In both controlled and free-living studies, when not correctly estimating steps, devices tended to underestimate values. Validity compared with criteria was the best for Apple Watch and Garmin, while the MPE values for Fitbit, Samsung, and Withings fell within ±3% on average. Within brands, devices appeared to vary, with Fitbit Classic tending to overestimate steps, while Fitbit Charge tending to underestimate steps; however, the variability observed could be attributed to differences in the number of comparisons for each device and in wear locations of the devices. Our findings are consistent with previous reviews [178].

In controlled settings across all devices, heart rate was accurately measured with only a very small tendency for underestimation. Heart rate validity was only sufficiently tested in Apple Watch, Fitbit, and Garmin devices. Heart rate measured by photoplethysmography is only available in relatively new commercial wearable devices. All of the brands measured heart rate to within ±3% on average in controlled settings. There were few studies examining the validity of heart rate measures in free-living conditions, but it appears that Fitbit devices may underestimate heart rate depending on activity intensity. All devices were within acceptable measurement error for heart rate. To our knowledge, this is the first systematic review to examine heart rate validity, and it appears that devices are able to measure heart rate within acceptable limits.

Energy expenditure estimates varied widely with less than 10% of estimates falling within acceptable limits in controlled settings. In many of the studies, there did appear to be a tendency for systematic over or underestimation. On average, only Fitbit measured energy expenditure to within acceptable limits, but there was wide variation around the estimate. Energy expenditure estimates also varied by model, with the Fitbit Classic underestimating the value considerably and Fitbit Charge HR overestimating the value. We hypothesize that Fitbit may provide the best, though still not acceptable, measure of energy expenditure because the algorithm employs a published equation for estimating resting metabolic rate [179]. To our knowledge, the other brands do not publish information about the energy expenditure estimates. There does not appear to be a relationship among more accurate estimates of energy expenditure in devices that include heart rate (Multimedia Appendix 7).

Interdevice reliabilities for steps, heart rate, and energy expenditure were all very strong. However, compared with validity studies, there were fewer reliability studies, and we were not able to conduct comparisons between brands or devices owing to small sample sizes. Sufficient data for intradevice reliability was only available for step count. The results showed considerable variability within the same device for step count for Fitbit Charge HR, Fitbit Surge, Fitbit Zip, and Garmin Vivofit, with five, five, one, and two comparisons, respectively.

### Future Research

Future research in this area should focus on the following three main topics: relevance and age of the devices tested, data acquisition from the devices, and algorithms used by companies. First, relevance of the devices is important. Owing to rapidly developing technology, the majority of the tested devices included in this review are now out of date or discontinued. The nature of the consumer technology market is such that updated product iterations are commissioned even before the original iteration of a device is released. For example, the newest Apple Watch included in the review is the Series 2 watch. The Series 5 watch was released in the fall of 2019. The results are similar for all devices and brands; the Fitbit Charge HR is a popular model for validity and reliability studies, likely because of its moderate price point (approximately US $150) compared with more expensive models (eg, Garmin Fenix 5, approximately US $500). Given the current device specialization, device relevance (eg, swimming or sleep-specific watches), and price difference between devices, continuing to conduct the types of reliability and validity studies reported here will be a challenge. The increasing pace of device release combined with device specialization makes this type of research challenging.

Second, few studies reported on how data were acquired from the devices. We believe this has implications for the scale of and usability of the data collected. For example, in order to collect data, we infer that some studies counted the steps recorded on the device in short time intervals instead of connecting the device to a platform after recording. Other studies exported and downloaded data from user accounts on the brand website, while others collected data from the brand API. Collecting data from the device API is the best and most scalable method for physical activity researchers when using wearable device data. In order to do so, we must develop interdisciplinary collaborations and open source tools to allow these data to be collected (eg, Open mHealth) [180].

Third, the algorithms used in consumer wearables are constantly changing based on sensor development and technological advances. Companies can update their devices’ firmware and algorithm at any time. When the device is synced, the firmware is updated. Feehan et al discussed the importance of firmware updates in their review [13]. While we believe this is important, it is clear that companies must be more open about the algorithms they are using to estimate steps, heart rate, and energy expenditure. Given the continuing release of new devices, firmware and algorithm updates to existing devices, and lack of availability of raw data, we believe researchers may need to shift focus from traditional reliability and validity research to studies that can provide open estimates for physical activity intensities or sleep standardized across devices. These studies will need to use device APIs and machine learning methods in collaboration with interdisciplinary teams in order to move the field forward.

### Limitations

Over the course of time that it took to complete this review, much has changed with market share, technology, and even research methodologies. Though the market share of companies was a large determining factor of what devices were included in this review, the consumer wearable market is volatile. On November 1, 2019, Google purchased Fitbit for US $2.3 billion, a massive shift for the consumer wearable device market [181,182]. Further to this limitation is the ever-changing nature of consumer technology. As Table 1 shows, many of the devices utilized in the studies included in this review are so out of date that they are no longer available on the market. There is some potential for bias when including only English language studies in systematic reviews. However, studies have shown that the effect may be small in general but may be difficult to measure for an individual systematic review [183,184].

### Conclusion

This systematic review of 158 publications included assessments of consumer wearable devices from nine brands (Apple Inc, Fitbit, Garmin, Mio, Misfit, Polar, Samsung, Withings, and Xiaomi), with a focus on the reliability and validity of the devices in measuring heart rate, energy expenditure, and step count. This review examined the validity of consumer wearable devices in free-living and laboratory settings and further highlighted results of the inter- and intradevice reliability of the nine consumer wearable brands. Among the studies included, Fitbit was studied the most and Xiaomi and Mio were studied the least. Apple and Samsung had the highest validity for step count, and Apple, Fitbit, and Garmin were accurate nearly 50% of the time. No brand fell within the acceptable accuracy limits for energy expenditure. Interdevice reliabilities for steps, heart rate, and calories were all very strong. Sufficient data for intradevice reliability were only available for step count, and the results showed considerable variability. There was no specific device or brand that involved a complete assessment across all measures, and no specific brand stood out as the “gold standard” in fitness wearables. This review highlights the validity and reliability of readily available wearable devices from brands and serves to guide researchers in making decisions about including them in their research. As new devices and models enter the market, up-to-date documentation can help direct their use in the research setting.

## Appendix

Multimedia Appendix 1 Search strategies.

Multimedia Appendix 2 Modified Consensus-Based Standards for the Selection of Health Status Measurement Instruments (COSMIN) risk of bias.

Multimedia Appendix 3 Substudies.

Multimedia Appendix 4 Study demographics.

Multimedia Appendix 5 Device models.

Multimedia Appendix 6 Criterion measures.

Multimedia Appendix 7 Energy expenditure mean absolute percentage error stratified by heart rate measurement on the device.

## Abbreviations

API: application program interface
COSMIN: Consensus-Based Standards for the Selection of Health Status Measurement Instruments
MAPE: mean absolute percentage error
MPE: mean percentage error
