# Supplementary material for: Reliability and Validity of Commercially Available Wearable Devices for Measuring Steps, Energy Expenditure, and Heart Rate: Systematic Review
Source: JMIR Mhealth Uhealth. 2020 Sep 8;8(9):e18694. doi: 10.2196/18694 (PMC7509623; doi:10.2196/18694)
Supplement: Multimedia Appendix 1 [file mhealth_v8i9e18694_app1.pdf]

SEARCH STRATEGIES

PubMed (searched 28 May 2019)

1. "Accelerometry"[Mesh]
2. acceleromet*[tw]
3. "Fitness Trackers"[Mesh]
4. "activity monitor"[tw] OR "activity monitors"[tw] OR "activity monitoring"[tw] OR "activity tracker"[tw] OR "activity trackers"[tw] OR "activity tracking"[tw]
5. "fitness tracker"[tw] OR "fitness trackers"[tw] OR "fitness tracking"[tw]
6. "step count"[tw] OR "step counter"[tw] OR "step counters"[tw] OR "step counting"[tw]
7. "wearable device"[tw] OR "wearable devices"[tw]
8. fitbit[tw] OR "garmin"[tw] OR "misfit shine"[tw] OR (misfit[tw] AND (phase[tw] OR flare[tw] OR ray[tw])) OR "polar loop"[tw] OR "jawbone up24"[tw] OR (jawbone[tw] AND up[tw]) OR (under[tw] AND armour[tw]) OR samsung[tw] OR geneactiv[tw] OR empatica[tw] OR mio[tw] OR amiigo[tw] OR xiaomi[tw] OR actigraph[tw] OR "apple watch"[tw] OR withings[tw] OR sensewear[tw]
9. 1 OR 2 OR 3 OR 4 OR 5 OR 6 OR 7 OR 8
10. "Comparative Study"[pt] OR "Validation Studies"[pt] OR "Evaluation Studies"[pt]
11. "Validation Studies as Topic"[Mesh] OR "Evaluation Studies as Topic"[Mesh:NoExp] OR "Reproducibility of Results"[Mesh]
12. valid*[tiab] OR compar*[tiab] OR accura*[tiab] OR reliab*[tiab]
13. 10 OR 11 OR 12
14. 9 AND 13
15. 14 AND 2000:3000[dp]
16. 15 AND "English"[la]

Embase (searched 28 May 2019)

1. 'accelerometry'/exp OR 'accelerometer'/exp
2. acceleromet*:ti,ab
3. 'activity tracker'/exp
4. "activity monitor*":ti,ab OR "activity tracker*":ti,ab
5. "fitness monitor*":ti,ab OR "fitness tracker*":ti,ab
6. "step count*":ti,ab
7. "wearable device*":ti,ab
8. fitbit*:ti,ab OR garmin:ti,ab OR "misfit shine":ti,ab OR "misfit phase":ti,ab OR "misfit flare":ti,ab OR "misfit ray":ti,ab OR "polar loop":ti,ab OR "jawbone up*":ti,ab OR "under armour":ti,ab OR samsung:ti,ab OR geneactiv:ti,ab OR empatica:ti,ab OR mio:ti,ab OR amiigo:ti,ab OR xiaomi:ti,ab OR actigraph:ti,ab OR "apple watch":ti,ab OR withings:ti,ab OR sensewear:ti,ab
9. 1 OR 2 OR 3 OR 4 OR 5 OR 6 OR 7 OR 8
10. 'comparative study'/de OR 'comparative effectiveness'/exp OR 'device comparison'/exp OR 'validation study'/exp OR 'evaluation study'/de OR 'measurement precision'/exp
11. valid*:ti,ab OR compar*:ti,ab OR accura*:ti,ab OR reliab*:ti,ab
12. 10 OR 11

13. 9 AND 12
14. #13 AND [2000-3000]/py
15. #14 AND [english]/lim

SPORTDiscus (searched 28 May 2019)

1. acceleromet*
2. "activity monitor*" OR "activity tracker*"
3. "fitness monitor*" OR "fitness tracker*"
4. "step count*"
5. "wearable device*" OR "wearable technology"
6. fitbit* OR garmin OR "misfit shine" OR "misfit phase" OR "misfit flare" OR "misfit flare" OR "misfit ray" OR "polar loop" OR "jawbone up*" OR "under armour" OR samsung OR geneactiv OR empatica OR mio OR amiigo OR xiaomi OR actigraph OR "apple watch" OR withings OR sensewear
7. 1 OR 2 OR 3 OR 4 OR 5 OR 6
8. valid* OR compar* OR accura* OR reliab*
9. #7 AND #8
10. #9 AND Limiters - Published Date: 20000101-20191231
11. #10 AND Narrow by Language: - english
