# Supplementary material for: Reliability and Validity of Commercially Available Wearable Devices for Measuring Steps, Energy Expenditure, and Heart Rate: Systematic Review
Source: JMIR Mhealth Uhealth. 2020 Sep 8;8(9):e18694. doi: 10.2196/18694 (PMC7509623; doi:10.2196/18694)
Supplement: Multimedia Appendix 2 [file mhealth_v8i9e18694_app2.pdf]

Modified COSMIN criteria used for Risk of Bias Assessment: Validity

**Design Requirements**	**Excellent**	**Good**	**Fair**	**Poor**
1. Was the percentage of missing device data given?	Percentage of missing data described- number of participants in device groups included in the analyses provided; relative to total number of participants in the study	Percentage of missing data NOT described- just total number of participants included in analysis (no individual group numbers), or no indication of how many including in the analyses at all (i.e. only the number of participants in the study is reported, no indication how many were included in the analyses and if any measures for the devices were missing)		
2. Was there a description of how missing data were handled?	Described how missing data were handled- describe explicitly why data is missing and how they dealt with the missing data statistically (i.e. only data with both devices, or all available data was included in the analyses)	Not described but it can be deduced how missing items were handled- do not state explicitly how missing data was dealt with, but can be deduced from table that less data was included in analysis for each group than total number of participants in study	Not clear how missing items were handled- no information, for example table show missing data but no explanation	
3. Was the sample size included in the analysis adequate?	Adequate sample size (≥ 100)	Good sample size (50-99)	Moderate sample size (30-49)	Small sample size (<30)
4. Can the criterion used or employed be considered as a reasonable ‘gold standard’?	Criterion used can be considered an adequate ‘gold standard’ (evidence provided)	No evidence provided, but assumable that the criterion used can be considered an adequate ‘gold standard’	Unclear whether the criterion used can be considered an adequate ‘gold standard’	Criterion used can NOT be considered an adequate ‘gold standard’ (i.e. self-reported time in activity is not a valid reference criterion)
5. Were there any other important flaws in the design or statistical methods of the study?	No other important methodological flaws		Other minor methodological flaws	Other important methodological flaws
6. For continuous scores: Were percent differences or BA plots or MAPE calculated?	Percent difference AND equivalency OR BA Plot OR MAPE%/SE of means, RMSE, CV, CCC	Percent difference only		No PD or way to calculate PD, but has other measures for accuracy (BA plot, MAPE/SE of means, RMSE, CV, CCC)
7. For continuous scores: Was an intraclass correlation coefficient (ICC) calculated?	ICC calculated and model or formula of the ICC is described	ICC calculated but model or formula of the ICC not described or not optimal. Pearson or Spearman correlation coefficient calculated with evidence provided that no systematic change has occurred	Pearson or Spearman correlation coefficient calculated WITHOUT evidence provided that no systematic change has occurred or WITH evidence that systematic change has occurred	No ICC or Pearson or Spearman correlations calculated

Modified COSMIN criteria used for Risk of Bias Assessment: Reliability

**Design Requirements**	**Excellent**	**Good**	**Fair**	**Poor**
1. Was the percentage of missing device data given?	Percentage of missing data described- number of participants in device groups included in the analyses provided; relative to total number of participants in the study	Percentage of missing data NOT described- just total number of participants included in analysis (no individual group numbers), or no indication of how many including in the analyses at all (i.e. only the number of participants in the study is reported, no indication how many were included in the analyses and if any measures for the devices were missing)		
2. Was there a description of how missing data were handled?	Described how missing data were handled- describe explicitly why data is missing and how they dealt with the missing data statistically (i.e. only data with both devices, or all available data was included in the analyses)	Not described but it can be deduced how missing items were handled- do not state explicitly how missing data was dealt with, but can be deduced from table that less data was included in analysis for each group than total number of participants in study	Not clear how missing items were handled- no information, for example table show missing data but no explanation	
3. Was the sample size included in the analysis adequate?	Adequate sample size (≥ 100)	Good sample size (50-99)	Moderate sample size (30-49)	Small sample size (<30)
4. Were there any other important flaws in the design or statistical methods of the study?	No other important methodological flaws		Other minor methodological flaws	Other important methodological flaws
5. For continuous scores: Was an intraclass correlation coefficient (ICC) calculated?	ICC calculated and model or formula of the ICC is described	ICC calculated but model or formula of the ICC not described or not optimal. Pearson or Spearman correlation coefficient calculated with evidence provided that no systematic change has occurred	Pearson or Spearman correlation coefficient calculated WITHOUT evidence provided that no systematic change has occurred or WITH evidence that systematic change has occurred	No ICC or Pearson or Spearman correlations calculated
