# Supplementary material for: Reliability and Validity of Commercially Available Wearable Devices for Measuring Steps, Energy Expenditure, and Heart Rate: Systematic Review
Source: JMIR Mhealth Uhealth. 2020 Sep 8;8(9):e18694. doi: 10.2196/18694 (PMC7509623; doi:10.2196/18694)
Supplement: Multimedia Appendix 3 [file mhealth_v8i9e18694_app3.pdf]

Scenarios where publications were split into sub-studies (demographics must be reported separately in publication):

1. Controlled and free-living data collected from two different sets of subjects
   a. Block (2017) x2
   b. Kooiman (2015) x2
   c. Lee (2015) x2
   d. Rowe (2019) x2
   e. Thorup (2017) x2
2. Two different activity paradigms conducted on two different sets of subjects (ex. overground ambulation with a group of subjects and treadmill ambulation with a subset of that group)
   a. Battenberg (2017) x2
   b. Huang (2016) x2
3. Single activity paradigm conducted on distinct groups of subjects with outcomes reported by group
   a. Different health statuses [Appelboom (2015) x2]
   b. Different chronic injury [Fulk (2014) x2]
   c. Different age groups [Modave (2017) x3]
