# Supplementary material for: Reliability and Validity of Commercially Available Wearable Devices for Measuring Steps, Energy Expenditure, and Heart Rate: Systematic Review
Source: JMIR Mhealth Uhealth. 2020 Sep 8;8(9):e18694. doi: 10.2196/18694 (PMC7509623; doi:10.2196/18694)
Supplement: Multimedia Appendix 4 [file mhealth_v8i9e18694_app4.pdf]

STUDY DEMOGRAPHICS

Author	Year	Substudy	Reliability	Validity	Energy Expenditure	Step Count	Heart Rate	Controlled Setting	Free-Living Setting	# of Participants	# of Male Participants	# of Female Participants	Age Group	Health Group	Age (years)	Age SD (years)	Weight (kg)	Weight SD (kg)	Height (cm)	Height SD (cm)	BMI (kg/m2)	BMI SD (kg/m2)	Location
Abt	2018		x	x			x	x		15	8	7	A	H	32	10	73.5	14.8	175	8			UK
Adam Noah	2013		x	x	x	x		x		23	13	10	A	H	26.65	7.55	69.56	13.76	170.57	11.12			CT, USA
Alharbi	2016			x		x			x	48	25	23	A	H	65.6	6.9					27.9	2.4	NSW, AUS
Alinia	2017			x		x		x		15	8	7	A	H									WA, USA
Alsubheen	2016			x	x	x		x		13	8	5	A	H	40	11.9	81	13.6	172.8	8.6	27	3.4	NL, CAN
An	2016			x		x		x	x	35	17	18	A	H	31	11.8	71.7	13.4	172.9	7.3	23.8	3.1	NE, USA
Appelboom	2015	1		x		x		x		27	13	14	A	NH	49								NY, USA
Appelboom	2015	2		x		x		x		10			A	H	28								NY, USA
Arch	2018			x		x		x	x	50			A	NH									DE, USA
Bai	2016			x	x			x		52	28	24	A	H							24.0		IA, USA
Bai	2017			x	x	x	x	x		39	23	16	A	H	32	11	72.7	12.8	171.7	9.5	24.7	4	IO, USA
Balto	2016			x		x		x		45			A	NH	46.7	10.0			169.4	10.7	26.6	6.1	IL, USA
Battenberg	2017	1		x		x		x		30	18	12	A	H	25.6	2.5	73.4	14	175.9	8.7	23.5	2.9	CA, USA
Battenberg	2017	2		x		x		x		20			A	H									CA, USA
Beevi	2016			x		x		x		14	9	5	A	H	29.93	4.93							Denmark
Benedetto	2018			x			x	x		15	7	8	A	H									Italy
Block	2017	1		x		x		x		61	17	44	A	NH	50	14.4							NH, USA
Block	2017	2		x		x			x	21	7	14	A	NH	54	11.4							NH, USA
Boolani	2019			x	x	x		x	x	120	74	46	A	H	21.4	3.7					25.1	5.8	NY, USA
Böselt	2017			x	x	x			x	20	17	3	A	NH	66.4	7.4					28.9	5.4	Germany
Boudreaux	2018			x	x		x	x		50	22	28	A	H	22.71	2.99	67.79	14.01	162.71	5.79	25.83	4.83	LA, USA
Brazendale	2019			x			x		x	19	10	9	C	H	8	1.8							SC, USA
Breteler	2019			x		x			x	30	16	14	A	H	40.4	10.6					23.8		Netherlands
Brewer	2017			x		x			x	50	9	41	A	H	28.1	9.12	70.6	17	165.3	7.1			USA
Brooke	2017			x	x				x	95			A	H	28.5	9.9	78.6	14.2	174.3	9.6	25.7	3.4	NE, USA
Bunn	2018			x		x		x		20	10	10	A	H	26.6	11.5	75.8	19.3	163	34	26.4	6.3	NC, USA
Bunn	2019			x			x	x		22	9	16	A	H	25.4	6.9	73.9	3.1	171	11			NC, USA
Burton	2018		x	x		x		x		31	11	20	A	H	74.2	5.8	75.2	14.8	168.9	8.6			Perth, AUS
Cadmus-Bertram	2017			x			x	x		40	20	20	A	H	49.3	9.5					25.1	3.9	WI, USA
Chandrasekar	2018			x		x		x		27	3	24	A	NH	69.2	8.8					28.3	5.6	UK
Chen	2016			x		x		x		30	15	15	A	H	21.5	2	60.7	8.3	167.6	8.9	21.5	1.9	Taiwan
Chow	2017			x		x		x		31	19	12	A	H	24.3	5.2					23.6	2.6	NSW, AUS
Chowdhury	2017			x	x			x	x	30	15	15	A	H	27	6	69.7	13.2	172	10	23.4	2.5	UK
Chu	2017			x		x			x	104	35	69	A	H	31.0		60.0		163.0		22.6		Singapore
Claes	2017			x			x	x		12	6	6	A	H	28	4.79	69.28	13.24	176.23	10.97	22.14	3.46	Belgium
Clevenger	2019		x		x	x			x	48	17	31	A	H	20.9						24.2		MI, USA
Collins	2019			x		x			x	15	5	10	A	NH	68	8					30	6	MA, USA
Daligadu	2018			x		x		x		20	18	2	A	NH	61.3	10.2	82.9	12.7	170	20	29	7.4	ON, CAN
Dannecker	2013			x	x			x		19	10	9	A	H	26.9	6.6	75.1	17.1	173	10	25.1	4.6	CO, USA
De Ridder	2019			x		x		x		30			A	H									Belgium
Degroote	2018			x		x			x	36	18	18	A	H	39.43	17.77	68.43	12.09	172.28	8.22	23	3.5	Belgium
DeShaw	2018			x		x			x	61	28	33	A	H	41.8	9.3	85.9	20	172	8.2	28.9	5.8	IA, USA
Diaz	2015		x	x	x	x		x		23	10	13	A	H									NY, USA
Diaz	2016		x	x	x	x		x		13	0	13	A	H	32	9.2					24.2	3.4	NY, USA
Dominick	2016			x		x			x	19	4	15	A	H	21.0	3.6	61.6	9.7	164.8	7.6	22.5	2.3	DE, USA
Dondzila	2016			x	x			x	x	19	14	5	A	H	24.6	3.1	86.5	19.2	175.2	11.3	28	3.8	MI, USA
Dondzila	2018			x		x		x		40	17	23	A	H	21.6	2					24.6	4.2	MI, USA
Dooley	2017			x			x	x		62	26	36	A	H	22.55	4.34	72.02	18.99	170	11	24.6	4.77	TX, USA
Duclos	2019		x	x		x		x		20	13	7	A	NH	53.9	10.8							QC, CAN
Ehrler	2016			x		x		x		21	9	12	A	H	34.5	15.7							Switzerland
Falter	2019			x	x		x	x		40	32	8	A	NH	61.9	15.2	79	16.2	171.1	9.3	27	5	Belgium
Farina	2018			x		x			x	25	13	12	A	H	72.5	4.9					27.4	3.8	UK
Ferguson	2015			x	x	x			x	21	10	11	A	H	32.8	10.2					26.4	4.2	SA, USA
Floegel	2017			x		x		x		99	29	70	A	H	78.9	8.0					28	6.5	NC, USA
Fokkema	2017			x		x		x		31	16	15	A	H	32	12					22.6	2.4	Netherlands
Fortune	2014			x		x		x		12	3	9	A	H	31						24.7	5.5	MN, USA
Fulk	2014	1		x		x		x		20	19	1	A	NH	40.3	11.6							USA
Fulk	2014	2		x		x		x		30	15	15	A	NH	61.6	10.4							USA
Gaz	2018			x		x		x		32	10	22	A	H	35.8	7.8	76.6	17.6	168.8	8.7	26.8	5.2	MN, USA
Gillinov	2017			x			x	x		50	23	27	A	H	38	12	73	16	171	11	25	3.5	OH, USA

STUDY DEMOGRAPHICS

Author	Year	Substudy	Reliability	Validity	Energy Expenditure	Step Count	Heart Rate	Controlled Setting	Free-Living Setting	# of Participants	# of Male Participants	# of Female Participants	Age Group	Health Group	Age (years)	Age SD (years)	Weight (kg)	Weight SD (kg)	Height (cm)	Height SD (cm)	BMI (kg/m2)	BMI SD (kg/m2)	Location
Gilmore	2018			x		x		x		40	14	26	A	NH	65	13							VIC, AUS
Gomersall	2016			x		x			x	29	3	26	A	H	39.6	11					25.9	5	QL, AUS
Gorny	2017			x			x		x	10	7	3	A	H	25.4	3.7					22.9	3.8	Singapore
Gruwez	2017			x	x	x			x	20	7	13	A	H	30	5							Belgium
Gusmer	2014			x	x	x		x		32	7	25	A	H	21.2	1.7	61.5	12.7	167.1	6.1			MN, USA
Haegele	2017		x	x		x		x		14	13	1	C	NH	15.4								VA, USA
Haghayegh	2019			x			x	x		35	18	17	A	H	25.1	10.6					22.4	3	TX, USA
Hamari	2017			x		x			x	26	15	11	C	H	9.6		34		140		22.1		Finland
Hargens	2017			x	x	x			x	22	7	15	A	H	30.8	11.4	73.1	17.4	169.4	8.6	25.3	4.4	VA, USA
Hergenroeder	2019			x		x		x		43	8	35	A	H	87	5.7	68.3	13.4	1.61	0.09	26.1	4.1	PA, USA
Hernandez-Vicente	2016			x	x	x			x	18	9	9	A	H	21	1.24	64.1	7.9	169.5	6.58	22.25	1.75	Spain
Hochsmann	2018			x		x		x		20	6	14	A	H	37.5		64.5		168		23.5		Switzerland
Huang	2016	1		x		x		x		40	30	10	A	H	23.9	2.8	63.4	9.7	172	7	21.4	2.5	China
Huang	2016	2		x		x		x		10	7	3	A	H	23.6	2.1	60.3	10.8	169	7	20.9	2.3	China
Hui	2018			x		x			x	12	7	5	A	NH	62.6	9.3							ON, CAN
Husted	2017			x		x		x		12	1	11	A	H	20.8	0.94							NE, USA
Hwang	2019			x			x	x		51	27	24	A	NH	44.4	16.6	64.5	9.1	163.1	16.4	23.7	3	Korea
Imboden	2017			x	x	x		x		30	15	15	A	H	49.2	19.2	79.2	15.5	174	8.9			IN, USA
Jo	2016			x			x	x		24	12	12	A	H	24.8	2.1	71.3	9.9	166	7			CA, USA
Jones	2018		x	x		x		x		30	12	18	A	H	34	8	71	16	171	12	23.8	7.64	VIC, AUS
Keating	2018			x		x		x	x	17	4	13	A	H	20.35	0.86					21.20	1.28	TX, USA
Kendall	2018			x	x	x		x		50	25	25	A	H	25.8	8.1	75.1	15.7	167.5	9	25.7	4.1	USA
Khushhal	2017		x	x			x	x		21	21	0	A	H	31.4	7.2					26.1	2.9	UK
Klassen	2015		x	x		x		x		43	30	13	A	NH	65	10.66							BC, CAN
Klassen	2017			x		x		x		21			A	NH	55	10							CAN
Kooiman	2015	1		x		x			x	56	18	38	A	H	32	9.9					22.9	2.1	Netherlands
Kooiman	2015	2		x		x		x		33	16	17	A	H	37	12.1					23.6	2.3	Netherlands
Koshy	2018			x			x	x		102	66	36	A	NH	68	15							VIC, AUS
Kroll	2016			x			x	x		50	26	24	A	NH	64								ON, CAN
Lamont	2018			x		x		x		33	21	12	A	NH	69	8							QLD, AUS
Lee	2014			x	x			x		60	30	30	A	H	26.4	5.6	67.9	9.1	171.1	6.2	23.1	2.7	NE, USA
Lee	2015	1		x		x			x	43	22	21	A	H	21.4	2.7	71.4	7.7	170.7	3.1	24.4	2.5	IL, USA
Lee	2015	2		x		x		x		37	19	18	A	H	20.9	2.0	71.3	9.6	170.8	5.2	24.3	2.8	IL, USA
Lee	2018			x	x			x		43	22	21	A	H	37.1	10.79	66	10.49	167.5	8.66	23.4	2.13	Korea
Leth	2017			x		x		x		22	11	11	A	H	31.1	8.03							Denmark
Mahendran	2016			x		x		x		15	8	7	A	NH	63.4	8.3							ACT, AUS
Middelweerd	2017			x		x			x	30	10	20	A	H	23.9	3.9	65.2	7.5	173.8	9.3	21.6	2.2	Netherlands
Modave	2017	1		x		x		x		21	10	11	A	H	26.2	5.1					23	2.7	FL, USA
Modave	2017	2		x		x		x		20	9	11	A	H	53.7	7					25.7	4.6	FL, USA
Modave	2017	3		x		x		x		19	5	14	A	H	70.9	4.3					27	5.4	FL, USA
Montes	2017			x	x	x		x		49	26	23	A	H	23.4	6.7	76.5	18.7	172.1	11			NE, USA
Montoye	2017			x	x	x	x	x		32	18	14	A	H	23.5	1.3	79.5	3	173	1.5	25.6	0.8	MI, USA
Mooses	2018			x		x			x	144	72	72	C	H									Estonia
Morris	2019			x	x			x		47	22	25	A	H	28.5	11.6					23.7	3.9	USA
Murakami	2016			x	x			x	x	19	9	10	A	H									Japan
Navalta	2018			x		x		x		20	12	8	A	H	22.2	5.8	71.2	14.4	174.6	9.9	23.2	3.2	NV, USA
Nelson	2016			x	x	x		x		30	15	15	A	H	48.9	19.4					26.3	5.2	IN, USA
Nuss	2019			x	x			x		20	9	11	A	H	23.5	2.96					22.9	2.23	CO, USA
O'Connell	2016			x		x		x		15	7	8	A	H	21.1	1.1					22.68	2.23	Ireland
O'Connell	2017			x		x		x		37	12	25	A	H	39	13.87					25	3.79	Ireland
Park	2014			x		x		x		40	20	20	A	H	38.6	13.7	77	14.85	169	7	27.2	5.2	TX, USA
Paul	2015			x		x		x	x	32	12	20	A	H	67.7	5.7							NSW, AUS
Pelizzo	2018			x			x	x		30	16	14	C	NH	8.21	3.09	31.3	15.3	132.2	23.3	20.5	5	Italy
Phillips	2015			x		x		x		50	5	45	A	H	84.2	7.1							MO, USA
Poojary	2018			x		x		x		62			A	H	27.9	5.1	69.4	14.5	168.5	7.7	24.3	4.5	India
Pope	2019			x			x	x		21	7	14	A	H	24.5	3.5	64.4	8.9	171	8.3			MN, USA
Powierza	2017			x			x	x		22	10	12	A	H	22	2	71.16	17.34	175.09	14.73			NC, USA
Pribyslavska	2018			x	x			x		34	23	11	A	H	25.8	4.9	76.6	18.8	171.7	8.4			USA
Price	2017			x	x			x		14	11	3	A	H	23	6	69.7	11.4	174.6	10.8			VIC, AUS
Prieto-Centurion	2016			x		x		x		12	5	7	A	H									IL, USA

STUDY DEMOGRAPHICS

Author	Year	Substudy	Reliability	Validity	Energy Expenditure	Step Count	Heart Rate	Controlled Setting	Free-Living Setting	# of Participants	# of Male Participants	# of Female Participants	Age Group	Health Group	Age (years)	Age SD (years)	Weight (kg)	Weight SD (kg)	Height (cm)	Height SD (cm)	BMI (kg/m2)	BMI SD (kg/m2)	Location
Reddy	2018			x	x		x	x		20	9	11	A	H	27.5	6	67.9	10.8	173.2	9.5	22.5	2.3	ON, CAN; OR, USA
Reid	2017		x	x		x			x	22	0	22	A	H	21.23	1.63	62.18	8.05			22.35	2.34	QC, CAN
Roos	2017			x	x			x		20	12	8	A	H	23.9	1.92	66.9	10.02	174	8			Switzerland
Rosenberger	2016			x		x			x	40	19	21	A	H	36								CA, USA
Rowe	2019	1		x		x			x	47	15	32	A	H	25.7								USA
Rowe	2019	2		x		x			x	24	1	23	A	H	28.3								USA
Rozanski	2018			x		x	x		x	37	24	13	A	NH	64.4	45	77.1	15.4	171	9.3			ON, CAN
Sala	2019			x		x		x		39	23	16	C	NH	9.6	3.42							USA
Sanudo	2019			x			x	x		45	45	0	A	H	24	4	72.2	5.8	177	5			Spain
Sasaki	2015			x	x			x		20	10	10	A	H	24.1	4.5	69.9	14.7	170.4	10.9	23.9	2.9	MA, USA
Schaffer	2017		x	x		x		x		24	14	10	A	NH	54	13.4							AZ, USA
Schmal	2018			x		x		x		22	11	11	A	NH	81	8					25.9	5.4	Denmark
Schneider	2016			x		x			x	87	39	48	C	H	12.11	0.54							CA, USA
Sears	2017			x		x		x		10	5	5	A	H	23.3	5.23	73.27	10.94	175.26	6.1			NC, USA
Sharp	2017		x	x		x		x		56	27	29	C	H	3.69	0.58							UK
Simpson	2015			x		x		x		42	11	31	A	H	73	6.9					26.1	4.6	BC, CAN
Šimůnek	2016			x		x			x	20	14	6	A	H	34	6.3					24.3	4	Czech Republic
Šimůnek	2019			x		x			x	185	89	96	C	H	15.9	1.9	61.3	11.3	171.1	8.4	20.7	2.7	Czech Republic
Singh	2016			x		x		x		10	8	2	A	H	30.8	6							SA, AUS
Smith	2019		x	x		x		x		32	21	11	A	NH	49.7	14	87.8	21.1	176.1	11.6	28.1	5.3	AZ, USA
Stackpool	2014			x	x	x		x		20	10	10	A	H	22	1.31	72	7.98	173.6	7.76			WI, USA
Stahl	2016			x			x	x		50	32	18	A	H	26.28	5.68	75.65	10.0	174.52	6.1	24.69	2.9	NE, USA
St-Laurent	2018			x		x			x	16	0	16	A	NH	32.7	4.2					27.4	4.4	QC, CAN
Storm	2015			x		x		x		16	10	6	A	H	28.87	2.65	72	9.2	175	9	23.5	2.3	UK
Støve	2019			x			x	x		29	17	12	A	H	29	9.4	73	10.5	175	9	23.7	2.2	Denmark
Sushames	2016			x	x	x		x	x	25	13	12	A	H	23.7	5.8	74.3	12.1	170.9	5.1	25.3	4.2	QL, AUS
Takacs	2014		x	x		x		x		30	15	15	A	H	29.6	5.7					22.7	3.0	BC, CAN
Tam	2018			x		x		x		30	15	15	A	H	32.1	8.66							Hong Kong
Tedesco	2019		x	x		x	x	x		18	7	11	A	H	69.4	2.7	71.4	4.33	167.9	5.4			Ireland
Thiebaud	2018			x	x		x	x		22	20	2	A	H	22	3	75.9	10.2	173	9			TX, USA
Thomson	2019			x			x	x		30	15	15	A	H	23.5	2.96					22.84	2.23	CO, USA
Thorup	2017	1		x		x		x		20	10	10	A	H	39	13.79	82	15.40	184	25.19	26	6.00	Denmark
Thorup	2017	2		x		x			x	24	22	2	A	NH	67	10.03	83	10.23	173	6.45	28	2.69	Denmark
Tophøj	2018		x	x		x		x		20	10	10	A	H	25.6	2							Denmark
Toth	2018			x		x		x		12	6	6	A	H	35	13	72.1	20.5	170.7	10.7	24.3	4.4	TN, USA
Treacy	2017			x		x		x		166	91	75	A	NH	80	11							NSW, AUS
Tully	2014			x		x			x	42	17	25	A	H	43	24							UK
Ummels	2018			x		x		x		130	55	75	A	NH	61.5	11.1					27.7	5.2	Netherlands
Van Blarigan	2017			x		x			x	22	22	0	A	NH	66						26.7		CA, USA
Veerabhadrappa	2018			x		x		x		71	47	24	A	H							25.7	3.8	PA, USA
Voss	2016			x		x			x	30	14	16	C	NH	13	2.2	50	16.7	155.5	12.3			BC, CAN
Wahl	2017			x	x	x		x		20	10	10	A	H	25.15	2.35	70.65	8.35	175.25	7.05			Germany
Wallen	2016			x	x	x	x	x		22	11	11	A	H	24.9	5.6	72.7	11.8	173.1	9.9			QL, AUS
Wang	2016			x			x	x		50	22	28	A	H	37	11.3					23.5	3.1	OH, USA
Wendel	2018			x		x		x		33	19	14	A	NH	65.48	9.39					25.05	3.94	MA, USA
Wong	2018			x		x		x		25	13	12	A	H	25.96	7.86	78.0	20.29	172.92	11.31	25.81	4.85	MD, USA
Woodman	2017		x	x	x			x		28	20	8	A	H	25.5	3.7			175.7	8.1	24.9	2.6	TN, USA
Xie	2018			x	x	x		x		44	23	21	A	H	22.3	2.15	60.9	7.04	167.7	4.99			China
Zhang	2019			x	x			x		30	17	13	A	H	20.2	1.7	74	13.9	171	10	25	3.98	PA, USA
