# Supplementary material for: Reliability and Validity of Commercially Available Wearable Devices for Measuring Steps, Energy Expenditure, and Heart Rate: Systematic Review
Source: JMIR Mhealth Uhealth. 2020 Sep 8;8(9):e18694. doi: 10.2196/18694 (PMC7509623; doi:10.2196/18694)
Supplement: Multimedia Appendix 5 [file mhealth_v8i9e18694_app5.pdf]

DEVICE MODELS

Brand	Model	Release Year	Model Status	Wear Location				# of Validity Comparisons			# of Reliability Comparisons			Total # of Comparisons		
				Wr	WH	LAF	To	HR	EE	SC	HR	EE	SC	Validity	Reliability	All
Apple	Watch	2015	outdated	x				58	26	30	7	0	0	114	7	121
Apple	Watch Series 2	2016	outdated	x				9	7	1	0	0	0	17	0	17
Fitbit	Blaze	2016	outdated	x				16	2	0	0	0	0	18	0	18
Fitbit	Charge	2014	outdated	x				2	11	21	0	0	1	34	1	35
Fitbit	Charge 2	2016	outdated	x				38	9	30	8	0	8	77	16	93
Fitbit	Charge HR	2015	outdated	x				52	37	76	0	0	7	165	7	172
Fitbit	Classic	2008	outdated		x	x		0	20	17	0	4	4	37	8	45
Fitbit	Flex	2013	outdated	x		x		0	28	109	0	2	15	137	17	154
Fitbit	Flex 2	2017	current	x				0	0	0	0	1	1	0	2	2
Fitbit	Force	2013	outdated	x				0	0	6	0	0	0	6	0	6
Fitbit	One	2012	outdated	x	x	x	x	0	36	160	0	3	19	196	22	218
Fitbit	Surge	2015	outdated	x				9	10	18	0	0	5	37	5	42
Fitbit	Ultra	2011	outdated	x	x		x	0	10	39	0	4	4	49	8	57
Fitbit	Zip	2013	current		x	x	x	0	7	194	0	0	6	201	6	207
Garmin	Fenix 3 HR	2016	outdated	x				0	4	0	0	0	0	4	0	4
Garmin	Forerunner 225	2015	outdated	x				10	5	0	0	0	0	15	0	15
Garmin	Forerunner 235	2015	current	x				14	0	2	0	0	0	16	0	16
Garmin	Forerunner 405CX	2009	outdated	x				0	0	1	0	0	0	1	0	1
Garmin	Forerunner 735XT	2016	current	x				0	0	3	0	0	0	3	0	3
Garmin	Forerunner 920XT	2014	outdated	x				0	5	6	0	0	0	11	0	11
Garmin	Vivoactive	2015	outdated	x				0	0	6	0	0	0	6	0	6
Garmin	Vivofit	2014	outdated	x				0	30	81	0	0	3	111	3	114
Garmin	Vivofit 2	2015	outdated	x				0	0	11	0	0	0	11	0	11
Garmin	Vivofit 3	2016	outdated	x				0	0	6	0	0	3	6	3	9
Garmin	Vivosmart	2014	outdated	x				0	0	14	0	0	0	14	0	14
Garmin	Vivosmart HR	2015	outdated	x				9	2	13	0	0	0	24	0	24
Garmin	Vivosmart HR+	2016	current	x				28	6	26	8	0	8	60	16	76
Mio	Alpha	2013	outdated	x				13	1	0	0	0	0	14	0	14
Mio	Fuse	2015	outdated	x				2	0	5	0	0	0	7	0	7
Misfit	Flash	2015	outdated		x			0	0	6	0	0	0	6	0	6
Misfit	Shine	2012	outdated	x	x	x	x	0	8	36	0	0	0	44	0	44
Polar	A300	2015	outdated	x				0	1	1	0	0	0	2	0	2
Polar	A360	2015	outdated	x				8	2	6	0	0	0	16	0	16
Polar	Active	2011	outdated	x				0	0	6	0	0	0	6	0	6
Polar	Loop	2013	outdated	x				0	7	27	0	0	1	34	1	35
Polar	M600	2016	current	x				0	0	1	0	0	0	1	0	1
Polar	V800	2014	outdated	x				0	7	1	0	0	0	8	0	8

DEVICE MODELS

Brand	Model	Release Year	Model Status	Wear Location				# of Validity Comparisons			# of Reliability Comparisons			Total # of Comparisons		
				Wr	WH	LAF	To	HR	EE	SC	HR	EE	SC	Validity	Reliability	All
Samsung	Gear 2	2014	outdated	x				0	0	4	0	0	0	4	0	4
Samsung	Gear S	2014	outdated	x				1	1	7	0	0	0	9	0	9
Samsung	Gear S2	2015	outdated	x				0	0	3	0	0	0	3	0	3
Samsung	Gear S3	2016	outdated	x				1	1	1	0	0	0	3	0	3
Withings	Pulse O2	2013	outdated	x	x		x	0	38	50	0	36	0	88	36	124
Withings	Pulse Ox	2014	outdated	x	x			0	12	34	0	0	8	46	8	54
Xiaomi	Mi Band	2014	outdated	x				0	1	1	0	0	0	2	0	2
Xiaomi	Mi Band 2	2016	outdated	x				0	0	5	0	0	0	5	0	5
