# Supplementary material for: Reliability and Validity of Commercially Available Wearable Devices for Measuring Steps, Energy Expenditure, and Heart Rate: Systematic Review
Source: JMIR Mhealth Uhealth. 2020 Sep 8;8(9):e18694. doi: 10.2196/18694 (PMC7509623; doi:10.2196/18694)
Supplement: Multimedia Appendix 6 [file mhealth_v8i9e18694_app6.pdf]

CRITERION MEASURES

Accelerometry	Electrocardiography	Indirect calorimetry	Chest Straps	Pulse Oximetry
Actical	ProComp Infiniti T7500M	COSMED K4b	Polar T31	Nonin PureSAT
Actigraph GT1M	Quinton Q4500	COSMED K4b2	Polar H7	
Actigraph GT3X	Zensor Intelesens	COSMED K5	Polar PS800CX	
Actigraph GT3X+	CardioSoft	Fitmate Pro	Polar H6	
Actigraph GT3X-BT	Quinton Q-tel RMS	Jaeger Oxycon	Polar T13	
Actigraph GT9X Link	SOMNOmedics GmbH	Metamax	Polar H3	
Actigraph wActisleep-BT	Prucka Electrophysiology	Metamax 3B	Polar WearLink 31	
Actigraph wGT3X-BT	COSMED Quark C12x	Moxus Modular system		
ActiHeart	Phillips IntelliVue	Jaeger Oxycon Mobile		
ActiTrainer	BedMasterEx	Jaeger Oxycon Mobile 5.0		
ActivPAL	Infinity Delta	Parvo Medics TrueOne 2400		
ActivPAL3	Biopac MP150	Sable System		
Bodymedia SenseWear	Quinton Q-Stress	Ultima CPX		
Bodymedia SenseWear Mini	GE Healthcare CASE			
Bodymedia SenseWear Pro				
Model X6-2mini				
New Lifestyles NL-1000				
New Lifestyles NL-2000i				
Omron HJ-112				
