# Supplementary figures and images for: Reliability and Validity of Commercially Available Wearable Devices for Measuring Steps, Energy Expenditure, and Heart Rate: Systematic Review
Source: JMIR Mhealth Uhealth. 2020 Sep 8;8(9):e18694. doi: 10.2196/18694 (PMC7509623; doi:10.2196/18694)

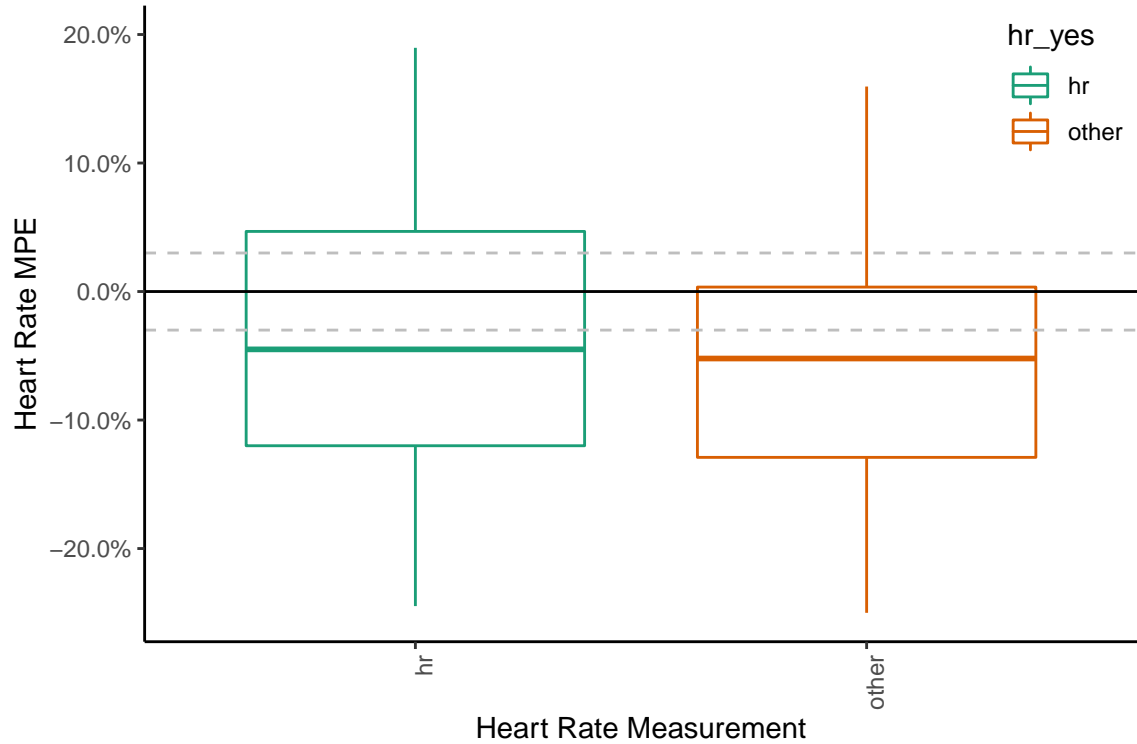

Supplement: Multimedia Appendix 7 [file mhealth_v8i9e18694_app7.pdf]
